# Cumulative social disadvantage and health-related quality of life: national health interview survey 2013–2017

**DOI:** 10.1186/s12889-023-16168-8

**Published:** 2023-09-04

**Authors:** Kobina Hagan, Zulqarnain Javed, Miguel Cainzos-Achirica, Adnan A. Hyder, Elias Mossialos, Tamer Yahya, Isaac Acquah, Javier Valero-Elizondo, Alan Pan, Nwabunie Nwana, Mohamad Taha, Khurram Nasir

**Affiliations:** 1 Division of Health Equity and Health Disparities Research, Center for Outcomes Research, Houston Methodist, Houston, TX USA; 2 Division of Cardiovascular Prevention and Wellness, Department of Cardiology, Houston Methodist DeBakey Heart & Vascular Center, 6550 Fannin St Suite 1801, 77030 Houston, TX USA; 3 Center on Commercial Determinants of Health, Milken Institute School of Public Health, The George Washington University, Washington, DC USA; 4 Department of Health Policy, London School of Economics and Political Sciences, London, UK; 5 Centre for Health Policy, Imperial College London, London, UK

**Keywords:** Health-related quality of life, Health and Activity Limitation Index, HALex, Quality of life, Social determinants of health

## Abstract

**Background:**

Evidence for the association between social determinants of health (SDoH) and health-related quality of life (HRQoL) is largely based on single SDoH measures, with limited evaluation of cumulative social disadvantage. We examined the association between cumulative social disadvantage and the Health and Activity Limitation Index (HALex).

**Methods:**

Using adult data from the National Health Interview Survey (2013–2017), we created a cumulative disadvantage index by aggregating 47 deprivations across 6 SDoH domains. Respondents were ranked using cumulative SDoH index quartiles (SDoH-Q1 to Q4), with higher quartile groups being more disadvantaged. We used two-part models for continuous HALex scores and logistic regression for poor HALex (< 20th percentile score) to examine HALex differences associated with cumulative disadvantage. Lower HALex scores implied poorer HRQoL performance.

**Results:**

The study sample included 156,182 respondents, representing 232.8 million adults in the United States (mean age 46 years; 51.7% women). The mean HALex score was 0.85 and 17.7% had poor HALex. Higher SDoH quartile groups had poorer HALex performance (lower scores and increased prevalence of poor HALex). A unit increase in SDoH index was associated with − 0.010 (95% CI [-0.011, -0.010]) difference in HALex score and 20% higher odds of poor HALex (odds ratio, OR = 1.20; 95% CI [1.19, 1.21]). Relative to SDoH-Q1, SDoH-Q4 was associated with HALex score difference of -0.086 (95% CI [-0.089, -0.083]) and OR = 5.32 (95% CI [4.97, 5.70]) for poor HALex. Despite a higher burden of cumulative social disadvantage, Hispanics had a weaker SDoH-HALex association than their non-Hispanic White counterparts.

**Conclusions:**

Cumulative social disadvantage was associated with poorer HALex performance in an incremental fashion. Innovations to incorporate SDoH-screening tools into clinical decision systems must continue in order to accurately identify socially vulnerable groups in need of both clinical risk mitigation and social support. To maximize health returns, policies can be tailored through community partnerships to address systemic barriers that exist within distinct sociodemographic groups, as well as demographic differences in health perception and healthcare experience.

**Supplementary Information:**

The online version contains supplementary material available at 10.1186/s12889-023-16168-8.

## Introduction

Patient-reported outcomes have gained traction in the evaluation of healthcare quality and monitoring of population health in recent years [1, 2]. These subjective outcomes directly inform providers and health policy-makers about the experiences of patients (or groups) and their value systems, both of which influence therapeutic choices, disease management, and health policy. Health-related quality of life (HRQoL), a commonly assessed patient-reported outcome, captures symptoms and functional limitations associated with health conditions and is known to be influenced by an individual’s psychosocial environment and their value-based preferences [3].

Likewise, the role of social determinants of health (SDoH) in perpetuating disparities in health and healthcare delivery has garnered attention as well [4–6]. SDoH are observed to influence objective health outcomes such as morbidity and mortality [7–9], and patient-reported health and wellbeing [10, 11]. SDoH are intricately interconnected and adverse determinants often cluster in marginalized groups [12–14]. Additionally, these proximal determinants can have differential impact on health which calls for a multidimensional framework in their assessment. Yet, evidence on the association between SDoH and HRQoL is largely based on single assessments of SDoH [11, 15, 16]. Very few indices of cumulative social disadvantage capture burden across an exhaustive range of SDoH domains [17, 18]. A comprehensive multidimensional SDoH framework may afford a provider or health system nuanced information to better identify at-risk individuals or those likely amenable to social interventions.

Thus, in this study, we utilized an exhaustive SDoH framework [19] to evaluate the relationship between SDoH and HRQoL, the latter assessed with the Health and Activity Limitation Index (HALex) [20]. HALex is a generic HRQoL measure combining self-reported health with the performance of activities of daily living and instrumental activities of daily living [20]. It summarizes a person’s HRQoL into a global score ranging from 0 (dead) to 1.00 (perfect health and functioning). We used HALex for the following reasons. Originally developed from the National Health Interview Survey (NHIS), HALex can be adapted to other national datasets as perceived health and activity limitation information are readily available [21–23]. A quality-of-life instrument with a global score such as HALex also minimizes interpretational challenges that may be associated with offsetting or contradictory changes to domain scores for multidomain instruments. Third, the simultaneous consideration of perception with physical functioning provides an incremental validity to HALex, unlike other generic measures which separately assess physical functioning [20]. We assessed HALex scores across levels of cumulative social disadvantage for the total population and by age, sex, and race/ethnicity. We hypothesized that higher levels of cumulative social disadvantage would be associated with poorer performance on HALex.

## Methods

### Data source and study sample

The NHIS is a cross-sectional household interview survey conducted annually by the National Center for Health Statistics [24]. A complex multistage area probability design is used to sample the non-institutionalized civilian population of the United States. Survey items are organized into the following core components: Household Composition, Family, Sample Child, and Sample Adult. The Household Composition core collects basic demographic and relationship information about all persons in the household. The Family Core, administered separately for each family in the household, collects information on sociodemographic characteristics, indicators of health status, activity limitations, injuries, health insurance coverage, and access to and use of health services. From each family, one sample child and one sample adult are randomly selected to gather further information on them. Additionally, NHIS has imputed income datasets to complete missing information on family income and personal earnings for each year. In this study, we merged the Household Composition, Family, and Sample Adult data files with the imputed income files.

Since the survey years 2013 to 2017 consistently featured items for our SDoH variables, we used data from these survey years to limit missingness. The data of respondents aged ≥ 18 years were merged and pooled over the five years. Survey weights were used to account for selection probability and non-response. NHIS data files are publicly available and deidentified, hence this study was exempt from the purview of the Houston Methodist’s Institutional Review Board.

### Study measures

#### Independent variable

**Fig. 1:**
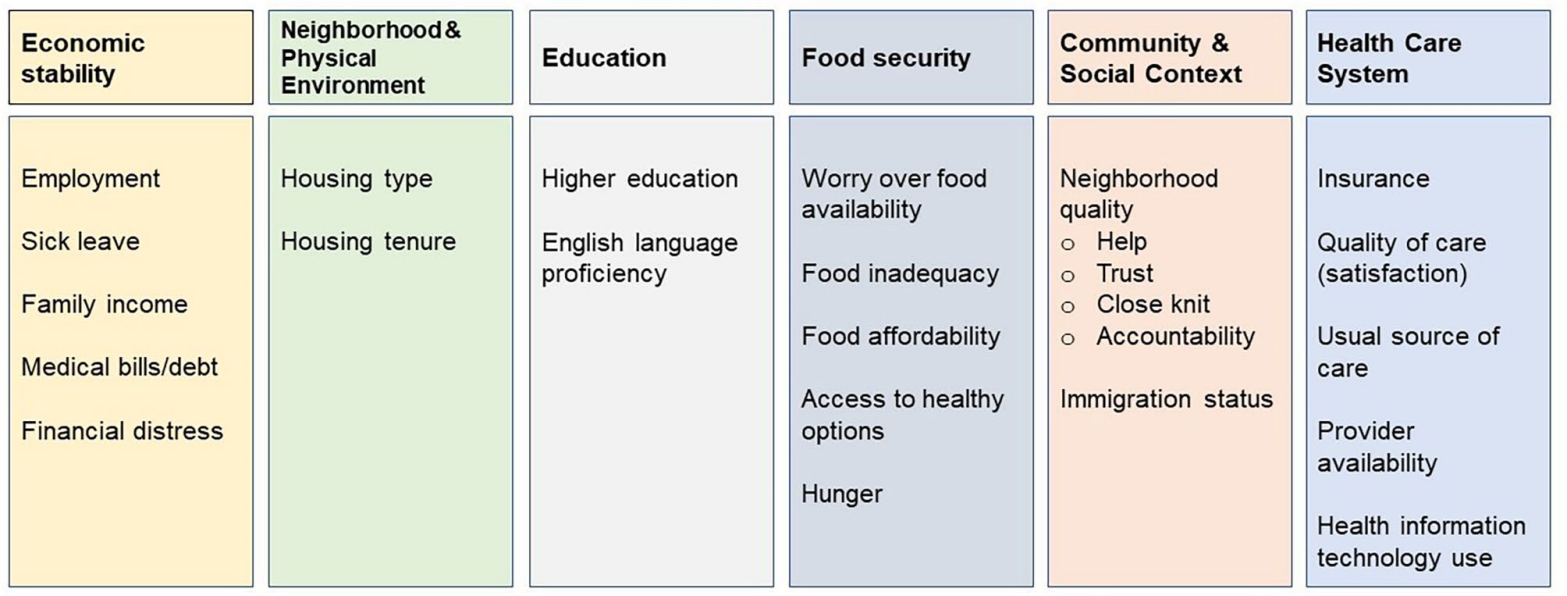
Social determinants of health from the National Health Interview Survey

Our cumulative SDoH index was developed by adapting the Kaiser Family Foundation SDoH framework of six SDoH domains [19]. Using responses from 47 survey items related to social factors, we organized SDoH into the following domains: economic stability, neighborhood and physical environment, education, food security, community and social context, and healthcare system (Fig. 1). Economic stability included employment, household income level, and financial distress from bills and debt. Neighborhood and physical environment included housing type and tenure. Education comprised higher education and English language proficiency. Food security comprised food availability, adequacy, affordability, and access to balanced meals. Community and social context was assessed with neighborhood cohesion, and immigration status. Healthcare system domain comprised insurance coverage, existence of a usual source of care, provider availability, and health information technology use. Each SDoH was scored as “0” if favorable and “1” if unfavorable. Supplemental Table S1 displays the specific survey items used to develop the cumulative index. Then, we ranked individuals by quartiles of the cumulative index. The first quartile group (SDoH-Q1) had the least cumulative social disadvantage, and the fourth quartile group (SDoH-Q4) had the greatest cumulative social disadvantage. We performed our primary analyses with both the continuous cumulative SDoH index and the quartile groups.

#### Dependent variable

HALex is a validated generic HRQoL measure developed from two attributes: perceived health status and functional activity limitation. For perceived health, respondents rate their overall health as ‘poor’, ‘fair’, ‘good’, ‘very good’, or ‘excellent’. For activity limitation, each person is classified into one of six categories of activity limitation based on age and the ability to perform a major activity, as shown in Supplemental Table S2. Categories of physical activity limitation include, “not limited in activities”, “limited in performing other activities”, “limited in performing a major activity”, “unable to perform a major activity”, “limited in instrumental activities of daily living”, and “limited in performing activities of daily living (i.e., personal care needs)” [20]. Persons who are classified into more than one category are assigned to the category with the greater degree of dysfunction. The responses to the two items are combined in a matrix of 30 health states. The most favorable combined health state is excellent health *and* no activity limitation, and the worst state is poor health *and* an inability to perform activities of daily living. A dead state is included as the 31st health state. Full details of the mathematical derivation of HALex – via corresponding analysis and a general multiplicative model based on multi-attribute utility theory – have been described elsewhere [20]. HALex scores range from 0.10 (the worst combined health state) to 1.00 (the most favorable combined health state) for persons alive. Dead state has a value of 0 [20].

We evaluated HALex as both a continuous outcome (HALex score) and a binary outcome, poor HALex (yes/no). Poor HALex was defined as a HALex score less than the 20th percentile (0.79) for the analytic sample [25]. Respondents (8.0%) with missing HALex values (i.e., with incomplete responses to at least one of the two attribute items for HALex) were excluded from the analytic sample.

### Covariates

Covariates included sex, age, race/ethnicity, region of residence (Northeast, Midwest, South and West), smoking status, psychological distress assessed with Kessler-6 score, and 11 self-reported comorbidities. Participants reported receiving a clinician diagnosis of arthritis, cancer, chronic liver disease, coronary heart disease (angina or myocardial infarction), chronic obstructive pulmonary disease, diabetes mellitus, hypertension, and stroke. They also reported a diagnosis of kidney failure in the twelve months before the interview. Finally, respondents recalled their weights and heights from which body-mass indices were estimated, and indices ≥ 30 kg/m^2^ were classified as obesity.

### Statistical analysis

We summarized respondent demographic and health characteristics, as well as selected SDoH variables, across quartiles of the SDoH index. Continuous variables were summarized with mean (SD) or median (IQR), where appropriate, and categorical variables were summarized with frequency and weighted proportion. Our summary of HALex scores and poor HALex were adjusted for age and sex. We compared adjusted marginal HALex means and poor HALex prevalence across SDoH quartiles in the overall study population, and by age, sex, and racial/ethnic groups.

For primary analyses, we evaluated both HALex outcomes first using the original SDoH index and then quartile groups of the index as independent variables. HALex scores were converted onto a decrement utility scale using negative linear transformation (X = 1 – U, where U = utility index) before assessment with two-part modeling [26]. We first modelled the probability that a person had a non-zero transformed score with a logit model using the full sample (first part). In the second part, we used an ordinary least square regression model to estimate the predicted difference in the transformed scores using the subset of people with non-zero scores [26]. To obtain predicted estimates and confidence limits on the original HALex scale, we simply back-transformed (1 – X) the regression estimates [26]. Poor HALex was analyzed using odds ratio (OR) estimates from logistic regression. For both outcomes, three sequential models were tested: an unadjusted model; Model 1 adjusted for age (continuous), sex, and race/ethnicity; and Model 2 further adjusted for smoking, comorbidities, Kessler-6 score, and region of residence. Further, we stratified our analysis of the outcomes by sex, age, and race/ethnicity categories.

We performed two supplementary analyses. First, we reanalyzed the cumulative SDoH-HALex association after multiply imputing by chained equations the missing values for variables with more than 5% missingness – close-knit neighborhood (5.2%), helpful neighbors (5.4%), trusting neighbors (5.6%), sick leave provision (6.4%), difficulty paying medical bills (7.6%), and English language proficiency (10.2%). Independent variables for the imputation models included all covariates in the full model of the primary analyses, HALex score, and the following SDoH-related variables selected with subject-matter knowledge – employment status, household income level, education, and insurance status. Twenty complete datasets were created. Secondly, we evaluated the independent associations between each SDoH and the HALex outcomes, adjusting for sex, age, race/ethnicity, and all the clinical characteristics.

All statistical analyses incorporated the complex survey design and weighting for selection probabilities and non-response. Variance estimation for the entire pooled cohort was obtained from the Integrated Public Use Microdata Series (https://nhis.ipums.org/nhis/). Statistical significance was assessed with a two-tailed significance level of 5%. We used Stata version 16 software (Stata Corp, College Station, Texas) for all analyses.

## Results

Table 1 describes the characteristics of the study population. The analytic sample included 156,182 NHIS adult participants with no missing HALex scores, representing 232.8 million adults in the U.S. (mean age 46 [SD 17] years, 51.7% women, 65.2% non-Hispanic White, and 15.8% Hispanic). The median cumulative SDoH index of the overall population was 3 (IQR, 2–6), with 37.7% in SDoH-Q1, 23.3% in SDoH-Q2, 16.6% in SDoH-Q3, and 22.5% in SDoH-Q4. Individuals in the more disadvantaged groups (higher quartiles of cumulative SDoH index) were younger, had higher representations of females, Hispanics and non-Hispanic Blacks, and a greater burden of comorbidities than their counterparts in the lower quartile groups. Supplemental Table S3 shows the distribution of the 47 SDoH across the quartile groups of the cumulative SDoH index. Supplemental Tables S4-S6 show the distributions of the combined health states across sex, age, and race/ethnicity groups.

**Table 1:** Descriptive characteristics of the adult respondents ≥ 18 years by quintiles of cumulative social determinants of health risk index: National Health Interview Survey 2013–2017

Characteristics	Total	Quartile groups of cumulative SDoH index			
		SDoH-Q1	SDoH-Q2	SDoH-Q3	SDoH-Q4
Cumulative SDoH score	3 (2–6)	2 (1–3)	5 (4–6)	8 (6–10)	13 (10–16)
Sample	156,182	55,758	36,459	26,881	37,084
Weighted sample	232,798,758	87,665,007 (37.7)	54,202,921 (23.3)	38,603,707 (16.6)	52,327,122 (22.5)
**Demographic characteristics**					
Sex					
Male	70,370 (48.3)	18,357 (49.9)	18,913 (48.0)	19,056 (48.0)	14,044 (46.3)
Female	85,812 (51.7)	29,703 (50.1)	20,092 (52.0)	14,813 (52.0)	21,204 (53.7)
Age, years	46.19 ± 17.41	50.83 ± 17.08	46.32 ± 18.29	42.95 ± 17.41	40.66 ± 15.11
Age group					
18–39 years	55,329 (39.5)	13,511 (28.7)	12,984 (40.3)	11,417 (47.1)	17,417 (51.2)
40–54 years	39,190 (26.8)	13,969 (27.8)	8,376 (25.2)	6,459 (25.2)	10,386 (27.7)
55–64 years	27,923 (17.1)	11,057 (19.5)	6,113 (16.2)	4,601 (15.7)	6,152 (14.8)
≥ 65 years	33,740 (16.6)	17,221 (23.9)	8,986 (18.2)	4,404 (12.0)	3,129 (6.2)
Race/ethnicity					
Non-Hispanic White	100,932 (65.2)	43,913 (78.5)	24,930 (68.1)	15,544 (58.0)	16,545 (45.2)
Non-Hispanic Black	20,411 (12.1)	4,704 (7.9)	4,515 (11.6)	4,456 (15.7)	6,736 (17.1)
Non-Hispanic Asian	8,934 (5.9)	3,097 (6.0)	2,325 (6.5)	1,735 (6.5)	1,777 (4.7)
Hispanic	23,906 (15.8)	3,587 (69.3)	4,223 (12.8)	4,742 (18.8)	11,354 (31.6)
Other	1,999 (1.0)	457 (0.6)	466 (1.0)	404 (1.0)	672 (1.4)
Region of residence					
Northeast	25,595 (17.8)	9,948 (20.1)	6,188 (18.3)	4,358 (16.7)	5,101 (14.2)
Midwest	33,826 (22.4)	13,108 (23.6)	8,319 (23.5)	5,624 (22.1)	6,775 (19.5)
South	55,595 (36.5)	18,896 (35.0)	12,339 (34.8)	9,692 (37.1)	14,668 (40.7)
West	41,166 (23.3)	13,806 (21.4)	9,613 (23.4)	7,207 (24.1)	10,540 (25.5)
**Health characteristics**					
Smoking history					
Never	94,162 (62.7)	26,998 (67.3)	18,945 (63.5)	20,755 (60.2)	16,178 (55.9)
Former	35,142 (21.4)	11,152 (24.7)	7,720 (22.3)	8,026 (19.3)	6,097 (16.5)
Current	26,300 (16.0)	2,972 (8.1)	3,399 (14.1)	5,467 (20.5)	6,103 (27.6)
Obesity	50,298 (31.9)	15,994 (28.3)	11,531 (31.6)	8,946 (33.5)	13,827 (37.0)
Arthritis	38,007 (21.6)	13,963 (22.3)	8,914 (21.6)	6,317 (21.0)	8,813 (20.9)
Cancer	14,386 (8.2)	6,759 (10.6)	3,367 (8.1)	1,975 (6.5)	2,285 (5.4)
Chronic liver disease	2,067 (1.2)	491 (0.8)	421 (1.1)	379 (1.2)	776 (1.8)
Coronary heart disease	9,923 (5.4)	3,295 (5.1)	2,366 (5.6)	1,721 (5.4)	2,541 (6.0)
Chronic obstructive pulmonary disease	5,258 (2.7)	1,215 (1.8)	1,177 (2.7)	1,066 (3.2)	1,800 (4.1)
Diabetes mellitus	15,168 (8.9)	4,645 (7.6)	3,488 (8.6)	2,847 (9.6)	4,188 (10.7)
Hypertension	50,647 (29.3)	18,799 (30.3)	11,983 (29.5)	8,471 (28.2)	11,394 (28.1)
Kidney failure	3,246 (1.7)	702 (1.0)	710 (1.7)	610 (2.0)	1,224 (2.6)
Stroke	4,581 (2.4)	1,218 (1.9)	1,009 (2.2)	899 (2.8)	1,455 (3.4)
Kessler-6 score (psychological distress)	1 (0–4)	0 (0–2)	1 (0–3)	1 (0–4)	3 (0–7)
**Social determinants of health (selected)**					
Low household income (< 200% FPL)	55,699 (30.7)	3,142 (4.4)	11,489 (25.6)	13,821 (45.5)	27,247 (69.2)
High financial distress*	57,650 (38.0)	7,215 (14.3)	12,041 (36.3)	12,265 (48.1)	26,129 (71.0)
Living quarters other than house/apartment/flat/condo	9,314 (5.2)	624 (0.9)	1,898 (4.2)	2,606 (8.3)	4,186 (11.0)
≤ High school/GED diploma	59,302 (37.4)	9,411 (16.2)	14,008 (38.2)	12,886 (48.8)	22,997 (63.6)
Not English-proficient	7,854 (5.6)	116 (0.2)	722 (2.5)	1,509 (6.6)	5,507 (17.4)
Food insecure†	16,746 (9.7)	186 (0.3)	1,178 (3.2)	3,100 (10.9)	12,282 (31.1)
Trust in neighborhood: somewhat/definitely disagree	26,102 (17.7)	416 (0.8)	2,586 (7.4)	5,447 (21.7)	17,653 (49.5)
Closeknit neighborhood: somewhat/definitely disagree	53,506 (36.1)	7,320 (13.8)	11,501 (34.6)	11,357 (46.0)	23,328 (65.7)
No insurance	19,030 (12.3)	301 (0.5)	1,541 (4.6)	3,616 (14.2)	13,572 (38.7)
No usual source of care	21,577 (14.1)	1,635 (2.9)	3,805 (10.7)	4,522 (18.0)	11,615 (33.4)

Notes: Mean (SD) or median (IQR) presented for continuous variables. Frequency (weighted %) presented for categorical variables.

All comparisons of study characteristics across quartile groups of cumulative SDoH index were statistically significant (p < 0.001)

* High financial distress includes the presence of ≥ 3 of the following: worried about money for retirement; worried about medical cost of illness/accident; worried about maintaining standard of living; worried about cost of normal healthcare; worried about paying monthly bills; and worried about paying rent/mortgage/housing cost

† Food insecurity includes the presence of ≥ 3 of the following in the last 30 days: worried food would run out before got money to buy more; food did not last before had money to get more; Could not afford to eat balanced meals; Cut size or skipped meals because not enough money, and if so, ≥ 3 days in the past month; eat less than felt should because not enough money; hungry but did not eat because not enough money; lose weight because not enough money for food; did not eat for a whole day because not enough money for food, and if so, ≥ 3 days in the past month

Abbreviations: *FPL* – federal poverty line; *GED* – general educational development; *SDOH-Q* – quartile groups of cumulative social determinants of health index

### Summary of HALex scores and poor HALex

**Fig. 2:**
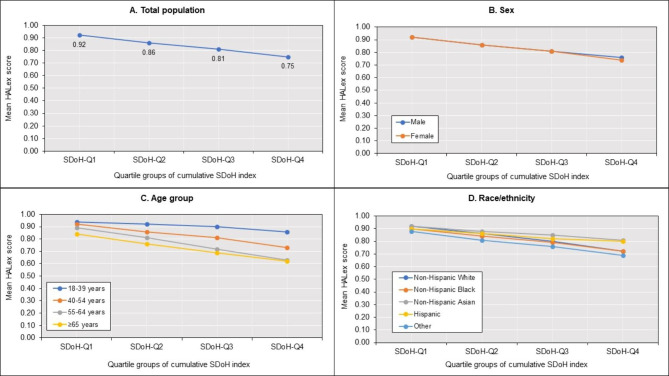
Age- and sex-adjusted marginal mean HALex scores. [Caption] (A) Total population. (B) Sex (C) Age group (D) Race/ethnicity. Abbreviations: HALex – Health and Activity Limitation Index; and SDoH-Q – quartile group of cumulative social determinants of health index

**Fig. 3:**
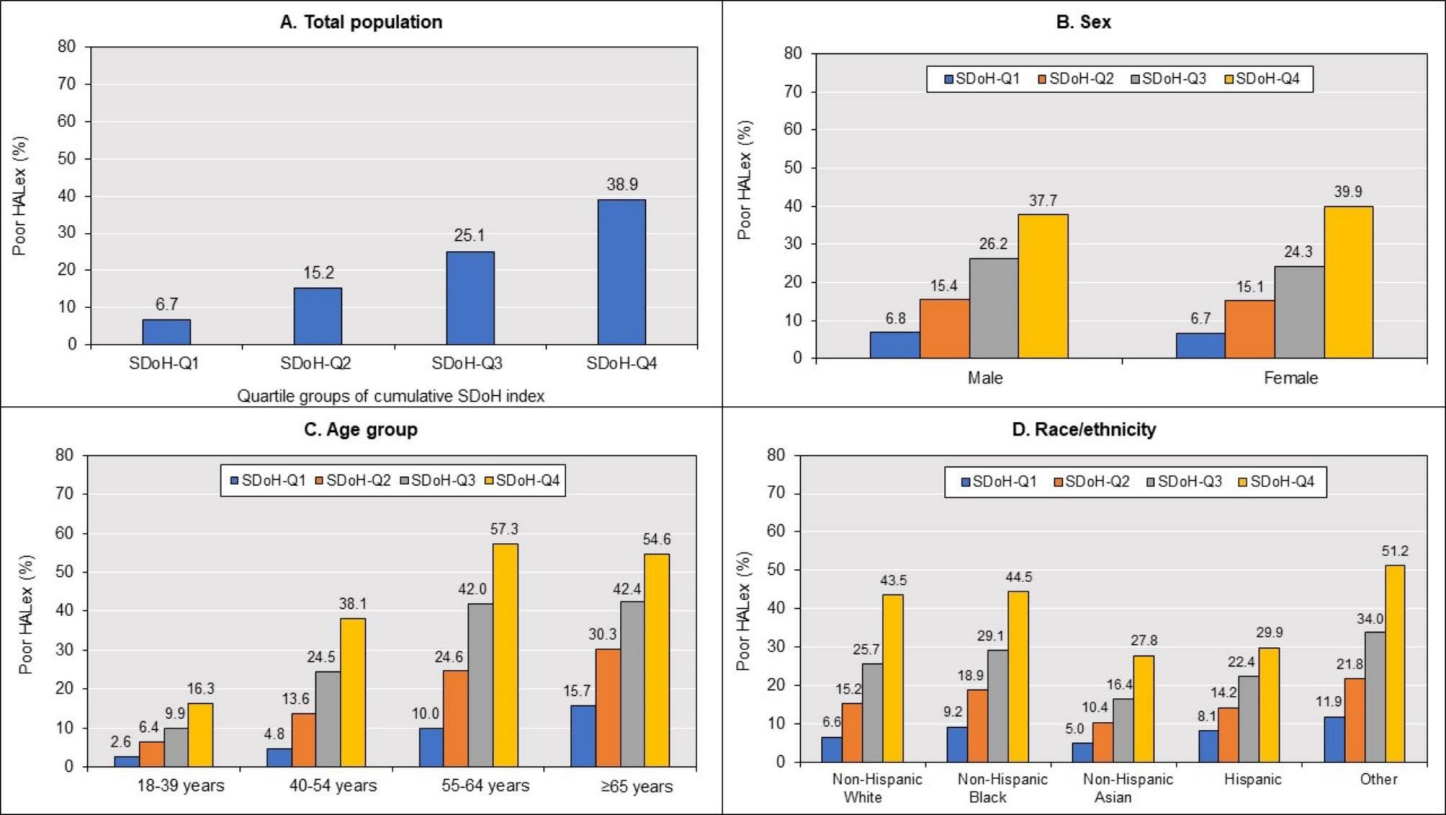
Age- and sex-adjusted prevalence of poor HALex. [Caption] Poor HALex score defined by HALex < 20th percentile score (0.79). (A) Total population. (B) Sex (C) Age group (D) Race/ethnicity. Abbreviations: HALex – Health and Activity Limitation Index; and SDoH-Q – quartile group of cumulative social determinants of health index

The mean HALex score of the overall population was 0.85 (SE 0.00), and 17.7% (95% CI [17.4, 18.0]) performed poorly on HALex. Adult males (0.85, SE [0.00]), adults aged 18–39 years (0.90, SE [0.00]), and non-Hispanic Asians (0.87, SE [0.00]) had the highest HALex scores (Supplemental Table S7). On the other hand, adults aged ≥ 65 years (0.79, SE [0.00]) and those of other racial/ethnic origins (0.78, SE [0.01]) had the lowest HALex scores. Figure 2 illustrates the mean HALex scores across SDoH quartile groups in the total population as well as across sex, age, and race/ethnicity categories. Overall, higher SDoH quartile groups had lower mean HALex scores. Men and women had similar HALex score profiles across cumulative SDoH quartiles. Adults aged ≥ 65 years had the lowest HALex scores across all cumulative SDoH quartiles, while those aged 18–39 years had the highest scores. Generally, non-Hispanic Asians had higher HALex scores than all other individuals at all levels of cumulative social disadvantage. At the highest level of cumulative disadvantage (SDoH-Q4), Hispanics had higher HALex scores than non-Hispanic Whites. Persons of Other origin, on the other hand, had the lowest HALex scores across all cumulative SDoH quartiles. Poor HALex was most prevalent in women (18.3%), individuals aged 55–64 years (27.3%), and adults of Other racial/ethnic origins (30.3%) (Supplemental Table S8). The prevalence of poor HALex increased with cumulative SDoH quartiles in the total population and across sex, age, and race/ethnicity groups (Fig. 3).

### Primary multivariable analyses

The results of our multivariable regression analysis are shown in Table 2. A unit increase in the cumulative SDoH index (i.e., an additional deprivation) was associated with 0.02 decrease in HALex score (β = -0.020; 95% CI [-0.021, -0.020]) after accounting for age, sex, and race/ethnicity. This difference was moderately attenuated in the full model (β = -0.010; 95% CI [-0.011, -0.010]). Using SDoH quartile groupings, predicted HALex scores decreased incrementally with higher cumulative SDoH quartiles, relative SDoH-Q1 in the minimally adjusted model (SDoH-Q4: β = -0.167; 95% CI [-0.171, -0.163]). This manner of HALex score decrements with quartiles of the cumulative SDoH index, persisted in the full model, albeit with some attenuation (SDoH-Q4: β = -0.086; 95% CI [-0.089, -0.083]).

**Table 2:** Association between cumulative social risk and the Health and Activity Limitation Index (HALex).

Independent variable	Unadjusted	Model 1	Model 2
A. HALex scores (β, 95% CI)			
SDoH index (1-unit increase)	-0.015 (-0.016, -0.015)	-0.020 (-0.021, -0.020)	-0.010 (-0.011, -0.010)
SDoH-Q1	Reference	Reference	Reference
SDoH-Q2	-0.044 (-0.046, -0.041)	-0.056 (-0.059, -0.053)	-0.032 (-0.035, -0.030)
SDoH-Q3	-0.081 (-0.085, -0.077)	-0.107 (-0.111, -0.103)	-0.061 (-0.064, -0.057)
SDoH-Q4	-0.128 (-0.132, -0.124)	-0.167 (-0.171, -0.163)	-0.086 (-0.089, -0.083)
B. Poor HALex (OR, 95% CI)			
SDoH index (1-unit increase)	1.20 (1.19, 1.21)	1.31 (1.30, 1.32)	1.20 (1.19, 1.21)
SDoH-Q1	Reference	Reference	Reference
SDoH-Q2	2.18 (2.06, 2.30)	2.79 (2.63, 2.96)	2.19 (2.05, 2.34)
SDoH-Q3	3.44 (3.26, 3.63)	5.71 (5.39, 6.07)	3.73 (3.48, 3.99)
SDoH-Q4	5.26 (5.00, 5.54)	11.11 (10.47, 11.79)	5.32 (4.97, 5.70)

Model 1 – age (continuous), sex, and race/ethnicity

Model 2 – Model 1 + smoking history, obesity, arthritis, cancer, chronic liver disease, coronary heart disease, COPD, diabetes, hypertension, kidney failure, stroke, Kessler-6 score, and US geographic region of residence

Abbreviations: β – beta coefficient; HALex – Health and Activity Limitation index; OR – odds ratio; SDoH-Q – quartile group of cumulative social determinants of health index

Regarding poor HALex, a unit increase in SDoH index was associated with 31% higher odds of poor HALex (OR = 1.31; 95% CI [1.30, 1.32]) in the minimally adjusted model. This association remained with minimal attenuation in the full model (OR = 1.20; 95% CI [1.19, 1.21]). Assessing with SDoH quartiles, we observed incrementally higher odds of poor HALex with higher cumulative SDoH quartiles, in both adjusted models, with some attenuation in the full model. For example, the odds of poor HALex performance in SDoH-Q4 was more than 11-fold the odds in SDoH-Q1 (OR = 11.11; 95% CI [10.47, 11.79]) in Model 1. In the full model, SDoH-Q4 was associated with at least 5-fold higher odds of poor HALex than SDoH-Q1 (OR = 5.32; 95% CI [4.97, 5.70]).

Table 3 shows the results of our stratified analyses for HALex scores and poor HALex. The graded negative association between cumulative social disadvantage and HALex was found to be consistent across sex, age, and race/ethnicity subgroups. The associations were stronger in the older age groups, non-Hispanic Whites, and non-Hispanic Blacks. On the other hand, the decrement in HALex scores at higher levels of cumulative disadvantage were lower in non-Hispanic Asian and Hispanic individuals. Men and women had similar gradients in the negative association between cumulative social disadvantage and HALex scores. The trends in the associations between cumulative disadvantage and poor HALex across the demographic groups were similar to those described for HALex scores, except the age groups, wherein the odds ratio of poor HALex associated with SDoH-Q4 in adults aged ≥ 65 years was lower than that of the younger adults 40–64 years.

**Table 3:** Association between cumulative SDOH risk and HALex-based outcomes, stratified by sex and race/ethnicity; National Health Interview Survey 2013–2017

Stratifying variable	Cumulative SDOH	HALex score (β, 95% CI)	Poor HALex (OR, 95% CI)
Sex			
Male	SDoH index*	-0.006 (-0.007, -0.006)	1.12 (1.11, 1.13)
	SDOH-Q1	Reference	Reference
	SDOH-Q2	-0.029 (-0.033, -0.025)	2.00 (1.79, 2.44)
	SDOH-Q3	-0.045 (-0.049, -0.042)	2.86 (2.59, 3.15)
	SDOH-Q4	-0.089 (-0.094, -0.084)	5.38 (4.83, 6.00)
Female	SDoH index*	-0.006 (-0.006, -0.006)	1.11 (1.10, 1.11)
	SDOH-Q1	Reference	Reference
	SDOH-Q2	-0.028 (-0.032, -0.024)	1.77 (1.61, 1.94)
	SDOH-Q3	-0.042 (-0.046, -0.039)	2.43 (2.24, 2.64)
	SDOH-Q4	-0.085 (-0.089, -0.080)	4.66 (4.25, 5.10)
Age group			
18–39 years	SDoH index*	-0.004 (-0.004, -0.004)	1.08 (1.07, 1.09)
	SDOH-Q1	Reference	Reference
	SDOH-Q2	-0.020 (-0.025, -0.016)	2.06 (1.69, 2.52)
	SDOH-Q3	-0.027 (-0.030, -0.023)	2.43 (2.06, 2.86)
	SDOH-Q4	-0.055 (-0.060, -0.050)	4.02 (3.39, 4.77)
40–54 years	SDoH index*	-0.006 (-0.007, -0.006)	1.12 (1.11, 1.12)
	SDOH-Q1	Reference	Reference
	SDOH-Q2	-0.019 (-0.024, -0.014)	1.86 (1.58, 2.18)
	SDOH-Q3	-0.033 (-0.038, -0.029)	2.46 (2.14, 2.83)
	SDOH-Q4	-0.087 (-0.093, -0.081)	5.20 (4.51, 6.00)
55–64 years	SDoH index*	-0.009 (-0.009, -0.008)	1.13 (1.12, 1.14)
	SDOH-Q1	Reference	Reference
	SDOH-Q2	-0.025 (-0.032, -0.019)	1.70 (1.46, 1.98)
	SDOH-Q3	-0.058 (-0.064, -0.051)	2.69 (3.04, 3.06)
	SDOH-Q4	-0.125 (-0.135, -0.116)	5.65 (4.91, 6.50)
≥ 65 years	SDoH index*	-0.010 (-0.011, -0.009)	1.12 (1.11, 1.13)
	SDOH-Q1	Reference	Reference
	SDOH-Q2	-0.048 (-0.055, -0.041)	1.79 (1.61, 1.96)
	SDOH-Q3	-0.066 (-0.074, -0.058)	2.25 (2.03, 2.50)
	SDOH-Q4	-0.122 (-0.137, -0.107)	3.73 (3.23, 4.30)
Race/ethnicity			
Non-Hispanic White	SDoH index*	-0.007 (-0.007, -0.007)	1.12 (1.11, 1.13)
	SDOH-Q1	Reference	Reference
	SDOH-Q2	-0.027 (-0.031, -0.024)	1.85 (1.70, 2.00)
	SDOH-Q3	-0.043 (-0.046, -0.040)	2.56 (2.38, 2.75)
	SDOH-Q4	-0.094 (-0.099, -0.089)	5.09 (4.67, 5.56)
Non-Hispanic Black	SDoH index*	-0.007 (-0.007, -0.006)	1.11 (1.10, 1.12)
	SDOH-Q1	Reference	Reference
	SDOH-Q2	-0.036 (-0.046, -0.026)	2.05 (1.63, 2.60)
	SDOH-Q3	-0.049 (-0.057, -0.042)	2.68 (2.23, 3.21)
	SDOH-Q4	-0.100 (-0.109, -0.091)	5.41 (4.46, 6.57)
Non-Hispanic Asian	SDoH index*	-0.006 (-0.007, -0.005)	1.14 (1.11, 1.16)
	SDOH-Q1	Reference	Reference
	SDOH-Q2	-0.029 (-0.039, -0.019)	1.58 (1.05, 2.38)
	SDOH-Q3	-0.039 (-0.047, -0.031)	3.19 (2.31, 4.39)
	SDOH-Q4	-0.069 (-0.082, -0.056)	5.02 (3.65, 6.90)
Hispanic	SDoH index*	-0.004 (-0.005, -0.004)	1.08 (1.07, 1.09)
	SDOH-Q1	Reference	Reference
	SDOH-Q2	-0.023 (-0.033, -0.014)	1.95 (1.44, 2.63)
	SDOH-Q3	-0.034 (-0.041, -0.027)	2.31 (1.80, 2.97)
	SDOH-Q4	-0.064 (-0.070, -0.057)	4.06 (3.21, 5.11)

* Estimate for 1-unit increase in cumulative SDoH index

Notes: HALex scores were assessed with two-part models. Poor HALex was assessed with logistic regression. Results of ‘Other’ race/ethnicity group not shown due to limited sample size

All models adjusted for age, sex, or race/ethnicity (where appropriate), smoking history, obesity, arthritis, cancer, chronic liver disease, coronary heart disease, COPD, diabetes, hypertension, kidney failure, stroke, Kessler-6 score, and US geographic region of residence

Abbreviations: β – beta coefficient; HALex – Health and Activity Limitation index; OR – odds ratio; SDoH-Q – quartile group of cumulative social determinants of health index

### Supplementary analyses

After multiply imputing missing values of the covariates, we found cumulative disadvantage-HALex associations similar to those described in the primary analysis (Supplemental Table S9). Evaluating individual SDoH, employment status, household income level, cost-related barriers to medical care, housing, food security, literacy, and transportation barrier to medical care were most strongly associated with poorer HALex performance (Supplemental Table S10).

## Discussion

In this nationally representative study of US adults, we demonstrated that the simultaneous accumulation of adverse socioeconomic factors across 6 domains of SDoH – economic stability, neighborhood quality, education, food security, social cohesion, and healthcare system – was associated with lower HALex performance in a graded fashion. This negative association persisted across sex, age, racial and ethnic groups, even after accounting for demographic and clinical characteristics. Hispanics, despite having a higher burden of cumulative social disadvantage, had a weaker SDoH-HALex association than non-Hispanic Whites. Employment status, household income level, cost-related barriers to medical care, housing, food security, literacy, and transportation barrier to medical care were most strongly associated with poorer HALex performance.

Functional activity level and perceived health status are important determinants of quality of life and health care resource utilization [27, 28]. Existing literature assessing the association between cumulative social disadvantage and HRQoL have two shortfalls the present study addresses. First, indices used to evaluate the association of cumulative disadvantage with health status have mostly been limited to a few SDoH items and domains [17, 29, 30]. Here, we utilized a more nuanced construct of cumulative social disadvantage encompassing objective and perceived measures of economic stability, language and literacy, housing, community and social environment, and the healthcare system access and experience. Secondly, health status studies have more often than not assessed perceived health status and functional activity limitation separately as they capture different aspects of health status [17, 31]. Yet, these two measures are correlated [32, 33], and using HALex maximizes this complex relationship in providing a global quality of life score that offers an opportunity to track changes in HRQoL.

We highlight the lower decrement in HALex scores and the increase in the odds of poor HALex relative to cumulative disadvantage found in elderly persons compared to younger adults. Similar age-related trends have been observed in studies that assessed HALex differences for chronic conditions like arthritis [22]. A potential explanation is the phenomenon that vulnerable or aging populations tend to report better subjective health due to lowered expectations rather than ‘actual’ better health [34]. This observation could also be due to the selective participation of healthier elderly individuals in the survey and/or the lower participation of healthier persons in the younger age groups.

Generally, historically marginalized non-Hispanic Black and Hispanic groups experience worse health outcomes than non-Hispanic White persons [35]. Despite greater Hispanic presence in the more disadvantaged groups, we found that Hispanics had better HALex performance than their non-Hispanic White counterparts at comparable levels of cumulative social disadvantage (Fig. 1). Furthermore, we found that the negative association between cumulative disadvantage and HALex was relatively weaker in Hispanics than the non-Hispanic White population (Table 3). These findings point to diminished health returns for Hispanics and concur with the theory of diminished returns for marginalized groups, including racial and ethnic minorities [36]. The theory posits that the health effects of socioeconomic and psychological resources may be differentially weaker in marginalized populations. Such unequal health returns of resources (or lack thereof) across racial/ethnic groups may stem from structural and environmental barriers, in particular the pervasive effects of structural racism and discrimination experienced by Hispanic, non-Hispanic Black, Asian, American Indian, and other underserved communities, which may mitigate the effects of socioeconomic resources and other SDoH on the health and wellbeing of these vulnerable subgroups.

An alternative reason for the apparently better HALex performance of Hispanic individuals at higher levels of social disadvantage could be the different perception of health across sociodemographic characteristics. The notion of health is influenced by experiences and expectations that differ across racial, ethnic, and socioeconomic characteristics [37–39]. Therefore, the impact of cumulative social disadvantage on perceived health status may be lower in individuals with a more resilient construct of health, emanating from cultural- and self-efficacy. Hispanic communities are particularly noted to have such resilience [40], which may play a role in their lower odds of reporting fair/poor health compared to non-Hispanic White counterparts [41]. In our study population, despite more non-Hispanic Whites (64.7%) reporting excellent/very good health than Hispanics (57.3%), there was a greater proportion of non-Hispanic Whites (5.5%) with at least limitation in a major activity who reported fair/poor health, than Hispanics (4.3%) with similar health states (Supplemental Table S6). This draws attention to the concept of community resilience and the need for population “wellness” interventions to be responsive to such phenomenon.

### Implications

There are several implications for our study results. First, the socioeconomic gradient found with such a patient-reported outcome that influences healthcare utilization and the value of care adds to the growing calls to integrate social care with clinical workflow [42]. This begins with improving patient-provider communication on health-related social needs and standardized screening and documentation of SDoH data. Despite the willingness of most patients to engage their providers on their socioeconomic circumstances during clinical encounters, such discussions often do not happen at all [43–45]. Clinicians can strategize to include concise conversations about SDoH in clinical encounters to better place patients’ expectations of their clinical care.

Regarding SDoH documentation, several screening tools [46, 47] and diagnostic coding [48] have been developed to enable hospitals capture the social needs data of patient groups. Software developers are also optimizing electronic medical records with user-friendly SDoH wheel to facilitate screening and referral to available community resources [49]. Standardizing screening and documentation of SDoH data will enable health systems to: track social needs more effectively in order to personalize care: identify population health trends: and guide community partnerships [48]. Unfortunately, adoption has been limited by a lack of clarity on who could document SDoH data, disincentivizing fee-for-service payment systems, and generally low prioritization by health systems.

Given the unequal return of socioeconomic and psychological resources across demographic divides, community-wide coalitions between health systems, public health agencies, and community partners present the most effective tool for addressing SDoH at both the community and individual patient level [50]. Such partnerships could better crystallize the unique social assets and risks of patient populations and inform the adjustment of clinical and social services to accommodate identified systemic social barriers.

### Study Limitations

We note some limitations of this study. HALex as a generic HRQoL measure is not without limitations. First, with the omission of emotional, mental, and social functioning from its derivation, HALex is limited in discriminating the levels of wellbeing, especially for populations who at the highest level of health [51]. Secondly, the reliance of HALex on subjective health status raises the issue of how much HALex score differences associated with socioeconomic disadvantage are related to differential reporting behaviors and health expectations. Beyond latent health, a person’s rating of their health is influenced by the interplay between their sociocultural environment and biology [39, 52]. Such heterogeneity may explain away some of the differences we observed with the concomitant HALex. Regardless, the manner in which people account for the many dimensions of health when rating their overall health is relatively stable across specific populations [37], assuring that the incremental negative changes in HRQoL associated with increasing cumulative social disadvantage are less likely to be artificial. Third, there is no established clinical minimally important difference for HALex, though a difference of 0.03 has been suggested as a threshold of clinical significance for health utility indices on which HALex derivation is based [40]. Nevertheless, HALex is strongly congruent with more widely used HRQoL measures [23, 53], and our results are consistent with studies that used other HRQoL measures [16, 54, 55].

Our cumulative SDoH index had some limitations as well. First, the self-report of all SDoH information without objectively assessed information may misclassify the social disadvantage profile of the study respondents. Nonetheless, previous studies have found a strong correlation between the self-reported information in NHIS, and the verified information found in other national datasets [56]. Secondly, simply adding up the SDoH items to quantify the overall burden of social disadvantage experienced across six domains may not sufficiently capture the potentially varying effects of individual SDoH on study outcomes. However, unweighted summation of individual social factors into an aggregate index is widely accepted [17, 30], and our approach for capturing SDoH burden in an aggregate index has been used to predict outcomes in diverse settings and patient populations [57–59]. A more thorough assessment of the relative effects of individual SDoH – within and across domains – on HRQoL and other patient-reported outcomes need to be explored in future studies. Finally, NHIS lacks objective measures of community-level disadvantage such as the area deprivation index or the social vulnerability index, for which reason our estimates may suffer some unmeasured confounding by community-level disadvantage. However, we have other community-context factors in our SDoH framework to provide some proxy for community disadvantage in our SDoH index.

## Conclusions

In a nationally representative study of US adults, cumulative social disadvantage was associated with poorer HALex performance in a graded fashion. In order to accurately identify socially vulnerable groups in need of both clinical risk mitigation and social support, innovations to incorporate SDoH-screening tools into clinical decision systems must continue. Policies can be tailored through community partnerships to address systemic barriers that exist within distinct sociodemographic groups, as well as demographic differences in health perception and healthcare experience, in order to maximize returns.

### Electronic supplementary material

Below is the link to the electronic supplementary material.

Supplementary Material 1

## Data Availability

The datasets analyzed in the current study are available from the corresponding author on reasonable request.

## Abbreviations

HALex: Health and Activity Limitation Index
HRQoL: health-related quality of life
NHIS: National Health Interview Survey
OR: odds ratio
SDoH: social determinant of health
SDoH-Q: quartile groups of cumulative social determinant of health index
